# The Hospital. Nursing Section

**Published:** 1905-12-16

**Authors:** 


### The Hospital.

### Nursing Section.

Contributions for "THE HOSPITAL," should be addressed to the EDITOR, "THE HOSPITAL" NURSING SECTION, 28 & 29 Southampton Street, Strand, London, W.C.

No. 1,003.—VOL. XXXIX. SATURDAY, DECEMBER 16, 1905. 8

### Notes on News from the Nursing World.

### OUR CHRISTMAS DISTRIBUTION.

ONE last word to any of our readers who may be able to assist in augmenting the number of articles which we hope to distribute next week among the hospitals and infirmaries most in need of warm clothing for the sick poor. There is still time to send in contributions, the final day being Saturday. We therefore hope to have to make some further acknowledgments of valuable and timely help. The whole of the articles received will be on view at the offices of THE HOSPITAL on Tuesday afternoon next, between 3 and 5 P.M.; nurses and other friends will be welcome. Since last week parcels are to hand from Nurses E. and G. B.; Private Nurse, St. Leonards; "One of the First Thousand" of the Pension Fund Nurses; Miss Evelyn Riches, member of the Royal British Nurses' Association; and Miss M. A. B. Groom, New Bond Street, W. All contributions should be addressed to the Editor, 28 and 29 Southampton Street, Strand, London, W.C., and should have "Clothing Distribution" written outside them.

### THE SCOTTISH BRANCH OF THE JUBILEE INSTITUTE.

THE annual meeting of the Scottish Branch of Queen Victoria's Jubilee Institute for Nurses was held last week at Edinburgh, Lord Salvesen in the chair. The report showed that the Council were responsible for the maintenance of forty-one nurses and probationers, and that during the year ending October 31, twenty-seven nurses had, on completion of their training, been engaged from the home by local committees. Two hundred and ninety-two inspections of single nurses and small homes were made during the year. There are now 268 Queen's nurses in Scotland, working under 174 affiliated branches. The general subscriptions amounted to £682, the subscriptions from district collections to £135 17s., and donations treated as ordinary revenue came to £531 13s. Lord Salvesen mentioned in his speech some further details, showing that the cost of the visits worked out roughly at sixpence or sevenpence each, and that this included the expense of training the nurses. He believed that the nurses had each on an average over the year 25 cases to attend to every day, and we are not surprised that he added they were overworked last year. He proceeded to state that, thanks to the liberality of a single contributor, the Council were in a position to engage an additional nurse. This is satisfactory as far as it goes, but Queen's nurses should not, in any circumstances, be required, as a regular thing, to attend so many as 25 cases daily.

### NEW MINISTERS AND NURSING QUESTIONS.

THE interest of the nursing world centres in the appointment of Mr. John Burns as President of the Local Government Board, and that of the Earl of Elgin as Secretary of State for the Colonies. Mr. Burns has, at any rate, shown that he appreciates the work of district nursing, and, but for being commanded by the King to kiss hands on his accession to office, he would have spoken on Monday at the annual gathering of the excellent body which does such good work in South London. There is scope for his official activity in another direction, if he will exercise his influence as head of the Local Government Board to secure the further elevation of the standard of nursing in Poor-law infirmaries. Lord Elgin, in the capacity of Colonial Secretary will have the opportunity of attesting his sympathy with the efforts to make better provision for the nursing of the sick in our dependencies across the seas. His immediate predecessor did not put himself to the trouble of even attending one of the meetings of the Colonial Nursing Association, but we trust that Lord Elgin will consider it worth while to follow the example set by Mr. Chamberlain and personally exert himself to extend the benefits of an organisation which could do with more vigour in administration.

### THREATENED STRIKE AT GARTLOCH ASYLUM.

WITH regard to the statements which have been made in some of the daily papers about a threatened strike of nurses at Gartloch Mental Asylum, the facts of the case are, we understand, as follows: The Glasgow District Lunacy Board has for some years been giving longer leave in its asylums than any other asylum in Scotland. It was decided a week ago to fall back on the leave previously in force, and instead of giving one whole day off duty per week and every third Sunday, to give one half-day a week and every third Sunday. This leave is as much as is given in any Scottish asylum at the present time. The staff of Gartloch Asylum naturally resented the change. The male staff sent to the Board a formal protest and a request for reconsideration. The female staff unfortunately sent an intimation of their intention to refuse duty on the morning on which shorter hours of leave were introduced. The strike, however, on more mature reflection, did not take place.

### THE RESPONSIBILITY OF NIGHT DUTY.

LAST week we advised the Battle Guardians to run the risk of having to put up with further changes in the nursing staff at the workhouse rather than leave the sick under their care without constant attendance at night. The importance of nurses being on duty in infirmary wards at night has since received another illustration. On Saturday night a fire broke out in the female sick ward at Tipperary Workhouse; fortunately it was discovered by the night nurse, with the result that help was at once obtainable and the flames were got under control. There were many patients in the ward at the time, all of whom, if not seriously ill, were in a weak state; and it is probable that if the night nurse at Tipperary Workhouse had been allowed to retire to rest, to be called only in case of necessity—as at Battle under the new conditions—there would have been a disastrous conflagration and several victims. Under any circumstances, night work involves a responsibility which can only be adequately discharged by the constant presence of the nurse on duty.

### PROVIDING A SUBSTITUTE.

In certain circumstances it is usual for the guardians of a union to ask a nurse who is obliged to be absent from her duties for a long time, to bear a portion of the cost of providing a substitute. This, in some cases, may be fair; but in that of a nurse whose absence is due to causes within the control of the guardians, it would clearly be unreasonable. It is therefore satisfactory that the guardians of Newport, Salop, have waived the condition in regard to their workhouse nurse, since it was shown at their last meeting that the medical officer attributed her illness to the bad air of the infirmary. It is hard lines that the nurse should have been incapacitated by the conditions under which her work had to be done, but to have insisted upon making her pay in cash for the deficiencies of the authorities would have been an intolerable injustice. We suggest that, in order to avoid any further instances of breakdown, the Newport Guardians should forthwith proceed to put the infirmary into a hygienic state.

### A HANDSOME GIFT TO HOSPITAL NURSES.

The staff nurses of St. Mary's Hospital, Paddington, are indebted to Messrs N. M. Rothschild and Sons for the possession of a piano by Broadwood. On Friday last invitations were given to the nurses to attend a gathering to be held in the staff nurses' room, which caused considerable excitement and curiosity amongst them. Upon entering the room they discovered the reason for which they were assembled. It was to celebrate, by a concert, the bestowal of a most acceptable piece of furniture. The piano is a very handsome instrument, and bears on the brass plate the names of the recipients and the donors. The matron provided tea, coffee, and cakes in honour of the event, and an extremely pleasant time was spent.

### CONCERT AT GUY'S HOSPITAL.

On Tuesday evening a concert was given at Guy's Hospital by the Nurses' Choral Society and the Students' Glee Club. Some charming part songs were sung, and there was also instrumental music, a nurse playing two excellent violin solos and a sister a 'cello obligato. Solos were sung by several of the nursing staff, and also by the chaplain and some of the students. There was a large and appreciative audience, and the pleasant evening finished up at 10 p.m., after refreshments had been served for the performers.

### BLACKBURN NURSES AND THE PENSION FUND.

A very encouraging number of nurses, secretaries, and members of committees of district nursing associations met in the Blackburn Town Hall last week in order to hear an address from Mr. Louis H. M. Dick, Secretary of the Royal National Pension Fund for Nurses. Invitations were sent out to hospital, district, and other nurses at Bolton, Preston, Darwen, Barnsley, Accrington, and neighbouring towns, several of which were represented at the meeting. Mr. John Rutherford, M.P., presided, and said that the Pension Fund was an absolutely safe means of providing at the lowest possible cost to the nurses themselves an allowance during incapacity for work caused by sickness or accident and a certain income for them in declining years. Mr. Dick, in the course of his address, set forth the objects and advantages of the institution, which, he pointed out, was not run for a profit, but for the benefit of the nursing profession. Several of the local clergy followed, and the Rev. J. P. Wilson said he thought that it was desirable that the district association which engages nurses should insure them. During the evening, refreshments were provided by the kindness of Mr. Pollick and Miss Dugdale, the Hon. Treasurer and Hon. Secretary of the Blackburn District Nursing Association.

### THE RECOGNITION OF THE CENTRAL MIDWIVES BOARD.

Persistence has been rewarded in the case of the Chorlton Guardians, who have just received an intimation that the Chorlton Workhouse Hospital has been recognised as a training school by the Central Midwives Board. According to Dr. Milson Rhodes, this decision has been obtained in spite of opposition by a Manchester medical man. However that may be it is satisfactory that the recognition has been accorded, both in this case and in that of the Belfast Maternity Hospital. In due time, no doubt, the claims of Aston Union Infirmary and other institutions hitherto excluded, will be allowed.

### THE CHURCH AND DISTRICT NURSING.

A course of sermons is being preached in the church of St. Dionis, Fulham, on the Wednesday evenings in Advent, on behalf of the Hammersmith and Fulham District Nursing Association. The Vicar, the Rev. W. S. Carter, D.D., who himself was one of the preachers, has emphasised the value of this excellent example by appealing to the members of the Sunday congregation who cannot attend the Wednesday services to send him contributions to be added to the offertories for the benefit of the association.

### A MODEL HOME FOR TORONTO.

The foundation-stone of the Nurses' Home in connection with the Hospital for Sick Children in Toronto has been laid by the eldest son of the generous donor, Mr. J. Ross Robertson. The estimated cost of the building is 75,000 dollars, but as

Mr. Robertson wishes it to be furnished and equipped in the most complete, not to say luxurious, manner, this sum will probably be considerably exceeded. The Home is to be erected as a residence for the nurses of the institution in memory of Mr. Robertson's first wife and her only daughter, and the function of laying the foundation-stone was attended by a large company, including a number of the nurses in their white and blue uniforms. In order that the Home shall be a model in every respect, the donor secured the best expert opinion of the Old World as well as the New before proceeding with it.

### VISITING NURSES IN MANCHESTER.

It is forty years since the Manchester and Salford Sick Poor and Private Nursing Institution was established, and at the annual meeting on Friday, the Lord Mayor, the Bishop of the Diocese, the Vice-Chancellor of Manchester University, and other notable persons were present. The record of work done in the last twelve months is admirable. The district staff, which now numbers 84, attended upwards of 10,000 patients and paid more than 245,000 visits—the largest number in a single year; while the private branch did so well that £125 was contributed out of its receipts to the general fund. The Bishop of Manchester said that the staff attached to the Institution comprised visiting nurses for the large class standing between the rich and the poor, who are often amongst the worst-off of our fellow-creatures. For their services they receive a small payment. It appears, therefore, that so far as Manchester is concerned, the problem of providing nurses for the needy of the middle classes has been solved.

### MIDWIFERY IN INDIA.

Dr. Louise Appel has gone to Benares City to set up in private practice among women. The undertaking is not lacking in the element of novelty or enterprise, and we hope that it will result, in course of time, in the better nursing of Hindu lying-in women. Dr. Appel has enjoyed the experience necessary for the important work which she has set herself to try and accomplish, but she admits that she does not expect in slow-moving India to do more than sow the seeds of reform.

### DISTRICT NURSING IN DEVONSHIRE.

The South Molton Nursing Association has just completed another year's work, during which the expenses amounted to £126 17s. 2d., the balance from 1904 of £8 12s. 10d., besides £10 from the Reserve Fund being practically absorbed. The Association, fortunately, has a good nest egg with £65 17s. 2d. on deposit. It was suggested that economy should be practised in the coming year, but Mr. Shapland advocated efficiency "without troubling about the money," and promised to assist in making up any deficiency. A district nurse is to be obtained for Clovelly, Woolfardisworthy, and Bucks Mills, and Mrs. Hamlyn presided the other evening over a meeting of 200 ladies from those parishes held at her residence, Clovelly Court. Miss Bell, Devon County Superintendent, spoke of the advantages to be obtained by having a trained nurse in the district, and the outcome of the discussion was that a strong committee was formed to obtain a nurse. It was agreed to affiliate the new organisation with the Devon County Nursing Association.

### THE FOUR "D's."

A welcome announcement was made at the eighth annual meeting of the Warrington District Nursing Association. The Chairman stated that since the report was published he had been able to get enough money to pay off the debt on the Nurses' Home, an amount between £900 and £1,000. Subsequently the report, showing that 20,244 visits were paid, and 519 cases nursed, was adopted. A deficit of £200 on the working expenses of the Association for the year was announced, notwithstanding that subscriptions and collections at places of worship had increased slightly, but the working men's contributions fell from £60 to £30. Two ladies had, unsolicited, organised a sale of work, the proceeds of which—£30—they had handed over to the institution, and had also sent a screen and an easy-chair for the use of the nurses. Miss Whitfield, the matron, in returning thanks for the kind things which had been said about the work, said as nurses they had to deal with four "d's"—drink, dirt, debt, and the devil; and, strangely enough, they themselves seemed to be suffering from the third of these "d's." We are glad to learn that there is a proposal to form a working man's committee, which seems badly needed.

### CO-OPERATIVE NURSING IN NICE.

We understand that Co-operative Nursing has begun this season at Nice, and that an association has been formed under influential auspices. Nurses in active work whose health is likely to benefit from a winter or two in the Riviera, may be glad to hear further particulars of this movement from Nurse Florence Warcup, who is willing to give any information desired.

### TRAINED MASSEUSES.

The next examination of the Incorporated Society of Trained Masseuses will be held on the following dates:—Written examination, Thursday, January 25th; anatomy and practical examinations, Friday and Saturday, February 9th and 10th, and Monday and Tuesday, February 12th and 13th. Application should be made to the Secretary, 12 Buckingham Street, Strand, W.C.

### SHORT ITEMS.

Miss May Fulford, eldest daughter of the Rev. R. M. Fulford, Vicar of Hennock, Devon, who has been nursing in South Africa for the past three-and-a-half years, has taken first place in the examination held for trained nurses by the Medical College of the Transvaal.—On behalf of an applicant for the vacant post of nurse at Tonbridge Union Workhouse, who appeared before the guardians the other day, it was urged that she possessed "an excellent alto voice, and would be an acquisition to any institution choir."—Miss Bessle's Comedy Company acted "The Comedy of Errors" for the entertainment of the in-patients of the Cancer Hospital, Brompton, on Monday evening. The different parts were very cleverly acted, and a hearty vote of thanks was accorded at the close of the piece.

### The Nursing Outlook.

"From magnanimity, all fear above;
From nobler recompense, above applause,
Which owes to man's short outlook all its charm."

### PRIVATE HOSPITALS AND NURSING HOMES.

In fact there is nowadays no difference between the private hospital and the nursing home. The Select Committee of the House of Commons made this clear in their report, where they state that nursing homes are deemed to include all homes and places conducted for profit where patients are taken in for treatment. Nursing institutions, on the other hand, are societies or bodies which supply nurses to the public. It will be remembered that the Select Committee declared it to be highly desirable that private hospitals and nursing homes should be licensed by the county or county borough authority within whose area they are situated, and that each such authority should require each of these establishments to conform to certain regulations and standards which it is desirable for them to promulgate. All regulations of the kind prepared by the county or county borough authority, when approved by the Local Government Board, were to be enforced by inspectors, who would have the right of entry and inspection. We have small doubt, as we said at the time, that Parliament will at once adopt the recommendations of the Select Committee in regard to these establishments, and that within a short time the existing abuses will be put an end to through the action of the county and municipal authorities referred to in the report of the Select Committee.

Since the report of the Select Committee was issued the New Zealand Government have introduced a Bill regulating private hospitals in that colony. It provides that every hospital of the kind must be licensed by the Government, and anyone who conducts such a hospital without a license is to be liable to a fine not exceeding £50. No license will be granted until the hospital has been approved by the inspector-general of hospitals and charities, and before granting a license the minister in charge must satisfy himself as to the character and fitness of the applicant. Every private hospital must have a manager who must be either a qualified medical practitioner or a registered nurse, in the case of surgical and medical hospitals, or a qualified medical practitioner or a registered midwife in the case of a lying-in hospital. The manager of each hospital will be required to give statutory notice of infectious diseases, births and deaths. Each private hospital must keep a register of patients. Every building of the kind must be open to inspection like ordinary hospitals, and it must only be used for the purpose for which it is licensed. Power is given to the governor in council to make regulations for the licensing, management, and inspection of all private hospitals, whilst the minister in charge has authority to cause an inquiry to be made into the conduct of any private hospital, and, if he thinks fit, to revoke its license. In those cases where a license has been revoked no new one shall be granted to the person in charge of such a hospital for five years at least. It is interesting to notice how closely colonial opinion seems to follow that of the Mother Country in questions which affect the well-being of the people at large. Here we have proof that experience points to identical remedies for existing abuses in regard to private hospitals and nursing homes. There can be no doubt, as ana nursing homes.

There can be no doubt, as matters stand at present, that the hygienic condition and the nursing arrangements of some so-called nursing homes leave much to desire. They are sometimes conducted without due regard to the necessities of the public and the claims of the patient. There is, too, the danger that the absence of any inspection or control of such establishments fosters the maintenance of certain homes, the continuation of which is not desirable in the interests of public morality, nor do they minister to the moral well-being of the people immediately concerned. It would be an immense gain if the county and municipal authorities were to set up a standard of hygienic efficiency, and to enforce its adoption by the managers of private hospitals and nursing homes throughout the country. As things are at present many a nurse has good cause to regret that she has invested her savings in some nursing home which in a few years has eaten up all her capital and left her to face the world once more with an empty purse.

Apart altogether from the protection which the license would afford to the public as represented by the patients who become inmates of private hospitals and homes, it could not fail to exercise a beneficial influence on the managers. Directly a local authority had formulated regulations and set up a standard, the average efficiency of such establishments must reach a point which would attract business, and so tend to protect the proprietors against loss. Such a standard must add greatly to the popularity of the private hospital in this country, and in a few years it is highly probable that public opinion would cause most paying patients and their friends to prefer treatment there rather than in their own homes. After all it is largely a case of supply and demand. Make all the private hospitals and nursing homes attain a high standard of efficiency, and they will soon become prosperous, for then they will be indispensable to the majority of intelligent people in the day of illness.

### Some Essentials in the Nursing of Infectious Diseases.

By A. Knyvett Gordon, M.B.Cantab.; Medical Superintendent of Monsall Hospital; Lecturer on Infectious Diseases in the University of Manchester.

It is within my experience that amongst nurses generally those who devote their time, or a portion of it, either to acquiring a knowledge, or to treating cases, of infectious disease, are apt to be looked down upon by their professional colleagues. I have also frequently observed that those who come to a fever hospital as sisters or senior nurses occasionally begin their career by showing this contempt, which is unwise, or by displaying a degree of incompetence, which is harmful.

Believing that this opinion is due to ignorance of the conditions that actually obtain in an isolation hospital, I propose in this paper to describe some of the general principles which underlie the nursing of infectious diseases, and to show, in a subsequent article, how these requirements can be carried out in practice.

Before doing so, however, it will be as well to consider for a moment how this contempt of fever nursing originated, and what place, if any, a course of instruction at a fever hospital should take in the training of the nurse herself.

I will say frankly that, like most widespread ideas, there is "something in it." That something is the fact—lamentable, it is true, but none the less a fact—that in some hospitals the training given to, and interest taken in, the nurse is wholly inadequate. This is again due to the custom that obtains in some of the larger fever hospitals of engaging the junior nurses for no fixed period, and the overloading of the matron's department with official duties (that could be done just as well by a clerk acting under her supervision) to such an extent that she can have but little time for personally supervising the ward work, and none at all to devote to getting to know the junior nurses.

In the smaller institutions it occasionally happens that the matron does not know how to, and the medical staff do not, train the nurses, either by precept or example.

To secure the best nursing it is, in my opinion, essential that the juniors should be engaged for a minimum period of two years, and that a certificate should be given at the end of this period. During this time every effort should be made to teach, firstly by actual supervision in the wards by competent sisters, and secondly by lectures, not only on practical nursing by the matron or her assistants, but also on anatomy, physiology, and the principles of infection, by the medical staff. To my mind the true function of such a hospital is to take nurses two years younger (*i.e.*, at the age of 21) than is customary at a general hospital, and to make every effort to place them as probationers in a general institution at the conclusion of their fever training. Fever hospital training alone does not fit a nurse to take general work, though I am sorry to say that it is within my experience that nurses possessing only a certificate of fever training have been accepted (without reference to the matron of the certifying hospital) as "fully trained nurses" in the less reputable variety of nursing home.

But to come to details. The first essential in the nursing of infectious disease is absolute asepsis, or, as I prefer to call it, surgical cleanliness. The degree of skill required in this respect is almost greater than in a general hospital, if only for the reason that the details of treatment are so much more in the hands of the nurse. Then, too, it is much more difficult to keep a septic case, in the midst of other septic cases, from getting more septic, than to keep out germs from an aseptic wound in the midst of other aseptic wounds. Yet it can be done, and I have seen it done time after time by the skill of the much-despised "fever nurse," and by nothing else.

To be aseptic it is necessary to understand what asepsis means. It means, in the first place, the realising that everything that has not been recently sterilised may be (and frequently is) a source from which germs can get into the patient's system, and set up fatal mischief there; and, secondly, the knowing how to keep these germs from getting to the patient, or how to kill them altogether.

Asepsis is seen in its simplest and easiest form in the operating theatre. By some nurses it is supposed to begin and end there; to such a one I would say, avoid fever nursing altogether, and take to, let us say, wheeling an elderly paralytic about in a Bath-chair.

No, it is necessary to give up altogether a somewhat senseless distinction between medical and surgical nursing. It is just as necessary to be aseptic about the nursing of a sore throat as of the radical cure of a hernia. The difference is that it is more difficult. Personally, though I am, I think, as strict as most people about details in the operating theatre, I am inclined, on the whole, to give as much credit for asepsis to the nurse who, in a ward containing 30 or 40 cases of scarlet fever, has her mild cases free from discharging ears and suppurating glands, as to her who hands me sterile swabs and threads my needles. I do not, of course, belittle the latter.

There are, however, two ways of being aseptic. One is to develop what I will call the aseptic instinct, and the other is to do it all as a matter of ritual. One is to feel it; the other to learn it off by heart. She who knows, without stopping to think, when anything is septic never makes a mistake. She who has learnt it off makes mistakes whenever she forgets, or is thinking of something else—that is to say, fairly frequently. In fact, the first is a good nurse; the second harmless, possibly, if well looked after.

The first essential for the attainment of the aseptic instinct is to understand, not merely that such and such things have to be done, but rather why they have to be; in other words, the nurse must have a good working knowledge of the principles of bacteriology—I do not say of its details. This she would obtain from the lectures in any well-organized fever hospital, and, incidentally, I strongly advise an aspirant to avoid any hospital where no such training is given, unless she takes up fever nursing as merely a temporary resort, and then I should not myself dignify it by the title of nursing at all.

The next requisite is experience, and here I very much doubt whether asepsis is ever attained instinctively in less than a year's hard work. Once attained, however, it will never leave her, however worried or "run down" she may be. It is worth striving for, for it is indeed a valuable possession for a young nurse to take with her to a general hospital at the end of her two years' fever work.

But to leave asepsis for a moment, there is another point about fever nursing, which is both a good and a bad thing, namely, that the work is physically harder in a fever hospital than in a general institution. It is bad, in that many who would otherwise make good nurses are unable to stand the severity of the work, and it is good in that those who can stand it are so much the better for the experience. The work, be it noted, is not therefore unhealthy; in fact, I often think it is healthier than the routine in a general hospital, because the wards are, as a rule, better ventilated and not so crowded in a fever hospital. But there are many patients to each nurse, and there are few cases that require so much manual labour for their proper care as children with bad attacks of scarlet fever or diphtheria.

(*To be concluded.*)

### The Nurses' Clinic.

### THE NURSING OF CHRONIC PATIENTS.

"Amongst other ailments we get a large number of ulcerated legs. The chronic type, I mean." These words were spoken to me by a district nursing superintendent with regard to the nature of diseases her nurses had to contend with.

"Chronic!" the very word makes one shudder. It set me thinking, too. Every district is not blest with a proficient number of trained nurses, and it is even possible that a busy country practitioner might occasionally be glad of outside help. Here, then, was a chance for the untrained woman worker, and perhaps the following suggestions might help her.

Chronic ulcers are frequently met with in the nursing world, and very often the doctor orders the Unna paste treatment to be carried out.

Unna's paste consists, amongst other ingredients, of gelatin. When cold it forms a thick gumlike elastic substance, and before using will need melting to the consistency of cream. For the process an ordinary glue pot or a double saucepan is generally used, but a pan of boiling water into which a second smaller receptacle can stand answers the purpose equally well. For the latter any clean tin, can, or jar large encugh to hold the required quantity will suffice, and it should be kept for the purpose. If the tin selected has no handle, the nurse could bore a couple of holes on either side and insert an impromptu one of plaited string or leather bootlaces, short and firm, so that it will not fall in the paste, but strong enough to bear the weight of the paste when it is lifted out to cool, or returned.

A fairly large paint brush will be needed, with soft firm hairs or bristles, substantial enough to hold a good dab of paste. If the hairs have become hard, through having previously allowed the paste to grow cold on them, putting the brush in with the paste when warming quickly renders them flexible. Whilst this preparation is going on the nurse will be making the patient ready for its reception.

The leg should be thoroughly washed with soap and water, and the ulcers and surrounding tissues gently cleansed with a swab of wool and some antiseptic solution (*i.e.*, boracic lotion), unless the doctor has given other orders. Then each ulcer must be dressed with a piece of medicated lint cut the exact size and shape of the wound. What ointment the lint will be spread, or lotion saturated, with the doctor, of course, will decide. But sometimes strong ointment is used, and if this is applied beyond the ulcer there is danger of it burning, or causing the healthy skin to deteriorate. The patient should be seated with the injured leg stretched out, and raised by resting the heel on a second chair. The floor and patient's clothes must be protected with something (clean old newspapers), for it is impossible always to avoid splashing with the paste, which is hard to remove when dry. Having dressed the ulcers and put the patient into position, the nurse should then ascertain that the paste is the desired consistency, and not too hot. If there is any doubt she should sweep the brush across the palm of her hand, for, of course, the paste in the centre of the brush contains more heat than that on the edge.

Now the first bandage will be applied. The bandages are made for this purpose of soft gauze, which is very adaptable. The chronic ulcer frequently attacks the inner side of the leg just above the ankle. In such a case it will be found convenient to fix the bandage by a turn over the instep, and then proceed with the figure of eight method to just below the knee, or as far as seems desirable. The bandage, of course, does not need, and should not have, a pin to fix it. The paste will now be painted on evenly, care being taken to reach and saturate the folds of bandage at the back of the leg over the calf. A slight turn of the leg on the patient's part makes this quite easy.

The paste must then be left for a moment to partially dry, then a second and a third bandage applied, unless special orders for more or less have been given.

There is need for careful and skillful bandaging; should there be a hitch or uneven twist under the coating of paste discomfort and irritation are caused and rest is banished. When dry and finished the whole should present a firm even case, capable of affording such support as will enable the patient to get about with comparative ease should circumstances prevent him taking absolute rest.

The length of time the bandages are allowed to remain on depends, of course, on the state of the ulcers. The doctor will say when he wishes them to be removed and then the patient must soak the leg over night in warm water, and have a temporary dressing applied so that the limb will be ready for examination when the doctor arrives in the morning.

Here, perhaps, the local treatment ends, but not by any means the nurse's duty. There is much still in a practical general way to be done.

Probably in these cases a tonic has been ordered; this suggests a lowered constitution, and that again the need of a liberal diet. Or the nurse may be greeted with such words as, "Doctor says he must be fed up, nurse, but we're pore people, we are, we can't get lux'ries, we can't." Certainly not, madam, but the doctor's idea of "feeding up" need not comprise a diet of mock-turtle soup varied with chicken and champagne. But good nourishing soup can be made from twopenny-worth of bones and a few vegetables.

Dabs can be bought for a 1d., and make a tasty dish if stewed in a little milk. 5½d. will buy tablets enough to make twenty-four junkets. A 5d. packet of cocoa will make a good many cups of that sustaining beverage. All these things need not be got at once, but if the head of the house can spare 6d., or even less, a week for the patient a good many extra dishes can be got out of it, which, added to the ordinary diet, will make a vast difference.

Fresh air is important; if it is "summer and fine weather" an armchair should be placed for the patient in a sunny spot, the diseased leg raised on another chair and protected from cold. If winter try and select a sheltered corner for him, and persuade him to have the window open.

*Warmth.*—Patients, especially if aged, suffering from chronic ulcers frequently have bad circulation, anything, therefore, which tends to aid it is advisable, and friction or massage of the extremities are beneficial. The patient should be as warmly clad as possible. Old people often feel cold even when sitting in the sun on a summer's day. Here one's thoughts fly to those benevolent old ladies (nearly every parish is blest with one or two) who delight to knit cuffs, gaiters, mufflers, waistcoats, shawls, and such like for "deserving cases."

In the nature of things chronic patients of any kind are prone to depression. No one admires "whiners," but on reflection there seems to be a good deal of excuse for them, and is it not possible to sympathise without encouraging? Often even their friends look upon them as a "burden," and their ailment a little thing of no account, because may be there is no intense pain, nor is it a sickness unto death. So often "chronics" suffer from what, to borrow the words of a novelist, has been spoken of as "the loneliness of introspection, the Gethsemane of the soul." In the night watches

THE NURSES' CLINIC—*Continued.*

when we are sleeping they very likely are watching with their burden, and in the day assume that indifferent mask which gives rise to such expressions as, "'e don't feel nothink 'e don't, nuss, 'e jest sits in that there corner all day grumbling or quiet like."

"How wags the world with you to-day, dad?" I asked an old friend and patient. "Saft and eaasy, miss; saft and eaasy," but (with a hasty glance round to make sure the "missus" was out of earshot) "times is when it's a gay scrap lonesome like." And as I trudged home I wondered just how much ground a "gay scrap lonesome" covered.

Ruskin, in one of his lectures with reference to "woman," said: "It is no matter of pride or perfectness in herself whether she knows many languages or one, but it is of the utmost that she should be able to understand the sweetness of a stranger's tongue."

One thing more; it seems well nigh impossible to nurse chronic cases with happy results unless the nurse be a bit of a philosopher. There is an atmosphere of healthy brisk philosophy in the proverb which says, "When God shuts a door He opens a window." But the invalid's outlook is proverbially, and in the nature of things, restricted. Yet doubtless he would take heart at the window if it was pointed out to him.

### Incidents in a Nurse's Life.

### THE CHRISTMAS LIGHT.

It was Christmas Eve in a London hospital. In the men's medical ward the lights were turned low. The two fires shed a warm glow on the bright red holly berries fastened over each bed.

The convalescents had been busy all day making paper shades for the gas globes and frills for the flower pots. Even those who were not well enough to be out of bed had carefully cut out decorations in pink tissue paper for the Christmas cakes. Now, the ward presented a truly frivolous appearance—quite unlike the usual neatness and order of every day. Garlands of pink roses hung in festoons from one side to the other. Little paper flags peeped out from unexpected places. The centre table had, beneath the deft fingers of Sister Elizabeth, been transformed into a veritable fairy land. It was covered with a soft layer of cotton wool, strewn with tiny sequins that glittered and twinkled like tiny stars. Soft satin ribbon of a pale green shade was twined artistically in and out round the small fairy lights, which were to be lit at 6 a.m., when the carol singers were expected. The flowers had not as usual been removed for the night.

Chrysanthemums of a golden brown tint filled the long, slender glasses, and a big bowl of Christmas roses, with their big moon faces, stood on the piano. Even the jug of the basin reserved exclusively for the use of the doctors had a bow of no ordinary size in green ribbon tied on to the handle.

The day nurses had long since gone off duty. Tired bodies and equally tired brains needed an extra amount of sleep to enable them to cope with the added work which Christmas Day involved.

The patients slept. Nurse Hope, in her noiseless round of the ward at 1 a.m., with a shaded lamp that she might scan each sleeper's face, smiled in a satisfied way as she passed bed after bed and heard only gentle, regular breathing. But when she came to the laryngitis case in the end bed by the fire she paused. Here was a patient very much awake. Two bright feverish eyes met hers with a defiance in them that mocked at slumber. A voice, parched with thirst, begged for a drop of water in husky tones.

"You were asleep both times I came round," she said softly, as she held the cup to his lips.

He shook his head. "I pretended I was so as you could get a bit of supper," he said in a hoarse whisper.

She filled the steam-kettle and propped him up with pillows. His breathing was slow and laboured. "Prap's if you was to stick that under piller in the 'oller of my back I'd go off," he said, with a weary sigh.

But when some time later she stole up to see if he was asleep she found his wakeful eyes fixed on the centre table.

"That tible's a fair picter—when are you goin' to light them night lights?" he asked.

"Not till six o'clock," she whispered.

"I want a clean 'andkerchief an' some lavender water on it," he said, with some difficulty, for his breathing was becoming more difficult.

"You are not feeling worse?"

She placed her fingers on his pulse.

"I can't breeve as easy. 'Taint nothink to get worryin' abart, but if I do die I'd like my 'andkerchief to be clean any 'ow," he whispered back.

Nurse Hope went back to her seat by the fire. She shivered slightly as she gazed into the glowing coals. The garlands of paper roses seemed to mock her. They spoke of a world beyond the hospital walls, where the endless struggle with disease and death was unknown. In thousands of homes she could see the great preparations being made for Christmas. Children's stockings bulged with presents; trees were being decorated with the skill of long practice. Squeaking lambs, celluloid golly wogs, tiny Father Christmases—they were all there. . . .

At 5.30 she made up the fires and roused the patients. They were all eager to look their best when the carol singers should come at six. Nurse Hope divided her attention between the laryngitis case and an old man with mitral disease. Number 10's breathing had become rapidly worse, and his voice was gone. The house surgeon, on being called up, suggested that tracheotomy might become necessary.

"'Ow long before they start singin'?" he asked her when the doctor had gone.

"Only twenty minutes." She paused to arrange his pillows.

He caught her wrist. "Ain't you goin' to light up soon?"

"Yes. In another ten minutes."

She passed down the ward with an armful of dressing-gowns. He lay and gazed at the table. The grey dawn lent an unearthly radiance to the shimmering cotton wool. He wondered vaguely why he had never thought before that it reminded him of piled-up masses of snow. Then he wanted to get out of bed and clear it away before Sister should come. He made a weak, ineffectual attempt to raise himself. And then the mist in his brain seemed to clear away. Someone put a cool hand on his forehead. When he looked up it was Nurse Hope. "I'm just going to light them," she said softly, with something in her face that vaguely puzzled him. "You've lit 'em," he whispered. "They're all of a twinkle, but it's gettin' dark over 'ere 'an—cold."

She held some brandy to his lips, but he pushed the glass away. "Keep quiet! I can 'ear 'em singin'," he said, holding up his hand.

. . . . . . . .

And afterwards she was glad to remember that Death had been very kind to Daddy. For he had seen the lights and heard the carol singing that no one else had heard.

### South London District Nursing Association.

In spite of a dense fog there was a large attendance at the annual meeting of the South London District Nursing Association on Monday afternoon. It had been arranged that tea and coffee should precede the speeches, which enabled those who had gone astray in the fog to struggle in by degrees in time for the more important part of the proceedings. Two disappointments awaited the audience, the first being that the Chairman, the Rev. Canon Erskine Clarke, was unable to put in an appearance.

The chair was accordingly taken by the Rev. W. J. Carey, who proceeded to announce the second disappointment, which was that Mr. John Burns would be unable to speak as he had promised, owing to his attendance being commanded by the King to kiss hands on his appointment as President of the Local Government Board. Mr. Carey said that Mr. Burns had told him that day how sorry he was not to be present, as he thought it would have been such a good send-off for him in his new office if he could have come straight away from the King to help in such a good cause as that of the Nursing Association. Mr. Carey then appealed to his hearers to transfer the enthusiasm they might have felt for Mr. Burns to the cause for which they were met together. The motives which actuated the nurses in their work of healing the sick and brightening their lives seemed to be two, the love of God and the love of humanity. After referring to some of the problems of the day, such as infant mortality, upon which subject he stated that Mr. Burns had intended to speak, Mr. Carey went on to mention the benefits the nurses conferred upon their patients by bringing so much brightness into their lives, and said how much the work of the nurses was appreciated and sympathy given them on all hands. A resolution that the meeting wished Mr. Burns all success and happiness in his new office was received with acclamation.

Dr. Wilson, who was the next speaker, bore testimony to the very high opinion in which the nurses were held by the medical men of the locality, and said that this Association improved in efficiency every year, excellent though it had been at the start.

The Rev. J. Cartwright alluded to the great debt of gratitude the Association owed to Canon Erskine Clarke, who had been so unfortunately prevented from being with them that day. Mr. Cartwright also spoke in feeling terms of the serious illness which one of the nurses had passed through. The proceedings terminated with the intimation that the total of the collecting cards was £55.

### Everybody's Opinion.

### IS THERE ANY ADVANTAGE IN JOINING THE PENSION FUND?

"Policy No. 4297" writes : I quite agree with all that is stated by Miss J. Anstey in your columns on December 2. I met with an accident while engaged as district nurse which has entailed two serious operations, since which I have been quite laid aside, and during the time (over a year) I have received £1 a week and great kindness from both doctor and secretary. I strongly advise all nurses to join the Royal National Pension Fund.

"Policy 3,920" writes : For the encouragement and help of doubtful or hesitating nurses I should like to add my testimony as to the great advantages to be gained by joining the Royal National Pension Fund for Nurses. My experience has been quite as encouraging as that of Miss J. Anstey. I am more than thankful I joined the Pension Fund ten years ago, for it has proved of the greatest benefit to me. I have been in ill-health on and off for about seven years, repeatedly having sick-pay, sometimes able for a time to take up my work again, but now have been receiving sick-pay regularly for a long period. I have always met with the greatest kindness and sympathy from the doctor, for which I am most grateful, as also for the kind consideration and courtesy shown me by the Secretary and all the officials. I wish I found it less difficult to persuade nurses to join our Fund, but it can only be because they do not fully realise the benefits of doing so.

. . We understand that relatively few nurses enter for sick pay. We think this must be due to insufficient knowledge. The extra premiums are small and the benefits they secure to each nurse are nothing less than the assurance that she may thenceforward be provided for for all her life if she arranges her policies on an adequate basis.—Ed. The Hospital.

### CHRISTMAS NOT ALWAYS A JOY.

"Another Matron" writes : It had been my intention for some months, prior to correspondence on the subject, to prohibit any present being made to me this year by my staff. I fancy that most matrons must have the same feeling, but perhaps think it would be ungracious and hurtful to the nurses to reject a present. I do not believe that such testimonials are ever subscribed to freely and ungrudgingly by all alike, and though it is nice to possess handsome pieces of plate and delicate articles of jewellery, for my own part I have pieces of needlework and inexpensive though pretty trinkets, which I prize far more highly than the costly presents because I know that they are the outcome of the sincere affection and esteem of those who have given them. I trust that many matrons will endeavour to prevent their nurses from imposing on themselves the heavy tax of Christmas presents, for there can be no doubt that it is a great tax at a season when there are so many more legitimate calls upon their oft-times slender purses.

"Nurse C. H." writes : Although much has been said respecting the burden nurses feel laid upon them in the way of presents and contributions at Christmas, I should like to put in a plea that those who send gifts of evergreens, clothes, toys, or money, for decorations to hospitals would remember the fever hospitals. While our general hospitals are so well remembered, our fever hospitals are forgotten, not only small isolation ones but large ones too, though the former are less frequently thought of. No doubt this is due to the fact that outsiders are so scared at the word "fever," that no such thing as a visit is possible, thus presents are overlooked. It is not generally known, perhaps, that the expense of decorations and presents in a fever hospital falls chiefly upon the sisters of the wards. No sister likes to feel herself behind, she is anxious to do her best to make her patients especially happy at this festive season and to give them a real good time. But all this means an outlay which a sister can often ill afford, and with the numerous collections for presents she frequently finds herself unable to do much for her own circle of friends and relations, and is truly glad when the expensive season is over. Matrons might help much if they would not only insist upon abolishing presents, but would also write a little paragraph to the local papers asking kind friends not to forget the "Fever Hospitals." I feel sure they would meet with a ready response, which would relieve nurses of a very great deal of expenditure.

[At the fever hospitals under the jurisdiction of the Metropolitan Asylums Board, presents from outside are not received.—Ed. The Hospital.]

### MENTAL PATIENTS. NIGHT AND NUDITY.

#### A Durham Scandal. Will the County Authorities Please Explain.

The Matron of the Durham County Asylum (Miss F. M. Mitchell) sends us a copy of a letter which she has addressed to the ratepayers of the county of Durham, through the medium of the local press : "Difficult as it may appear to comprehend that such injustice could be meted out to any Matron, yet I, who have experienced it, can vouch for the truth of this statement, and, after reading it, I respectfully leave it to the ratepayers to ask an explanation from those who are responsible for the management of this Asylum. One of our medical officers made a formal complaint against the stores' officials, who had neglected to supply articles of clothing due to the wards, which had been requisitioned and signed for by the medical superintendent many times, though no notice of his request had been taken by those responsible for the issuing of such goods. These requisitioned articles had been owing to the wards, in some cases, for nearly a year. So great was the strain becoming on the resources of these wards that many of the patients had to be put to bed unclothed, and many more, during the day, had to be deprived of necessary articles of clothing in order to add to the garments of those even less imperfectly clothed than themselves. I may mention that personally I had grown weary of placing written reports touching on the same important subject in the medical superintendent's office, which never received the smallest attention, and the charge nurses, as well as myself, had come to the conclusion that our grievance was a standing one, and we saw no chance of its being righted. Matters, however, came to a climax when the medical officer went to examine a patient and found she was without the garment very necessary to her comfort—a nightdress. He came to me for an explanation, as the bedding of any patient in such a condition seemed to him shameful negligence on the part of the nurse. I then explained that I had known this state of affairs existed for many months, and that there were many more patients in different parts of the house who had to be put to bed in a similarly unclothed condition. In the month of August I was sent for by the Committee of Visitors at their monthly meeting, when I answered questions supporting the medical officer's just complaint. After the meeting I met the medical superintendent in the grounds. He informed me that I, along with the medical officer who had drawn the Commissioners' and the Committee's attention to the grave complaint of the charge nurses whose patients were suffering, had been dismissed, but that he had asked the Chairman to allow us to resign. I told the superintendent that I would not resign—that I had supported the truth, and I wrote to the Chairman and told him the same, and I asked him why I was to be dismissed. He replied 'that I had imagined a state of affairs to exist which did not exist, and that, for the good of the asylum, I must resign.' Yet, as subsequently happened at an inquiry made by the Chairman, in the presence of no other member of the Committee, the charge nurses, the work-room books, the very patients themselves who had suffered, and the auditor's figures, all proved my answers to the Committee's questions to be true, and I still refused to resign. In the month of October the Chairman told me the Committee were at one, as they were in August, that my services as Matron were not to be retained. I then asked if I might have a reason, but the Chairman told me there was no special reason. Yet I was asked in August to resign, because I had 'imagined,' and, now that my 'imagination' had been proved to be the stern truth, there was no more said about it—not even an apology tendered to me after accusing me wrongly, and judging me without a hearing. Before handing me the document terminating my engagement here 14/1/06, the medical superintendent informed me he had been forced to sign my dismissal against his will, that he did not approve of the action of the Committee, and that he had no fault to find with me as Matron. In handing the medical officer a similar document, so convinced was the medical superintendent that he had done wrong in putting his signature to a dismissal he felt was unjust, that, in the presence of witnesses, he took a pen and drew a line through his signature, which, he said, had been forced from him by the legal adviser of the Committee, and with whom he had argued for ten minutes before signing it. At a subsequent meeting the superintendent was told he must sign another document, and this second document has accordingly been signed, with no explanation. After training as a hospital nurse, after ten years' experience as a Matron, five and a half years in that position having been spent here, I find myself in all the vigorous interest of my nursing career suddenly debarred for ever (through no fault of my own, but supporting the truth) from the calling I have chosen and made my life's work. A similar fate has befallen the medical officer. I respectfully repeat that I leave it to you as ratepayers to inquire further into this monstrous injustice."

### Appointments.

[No charge is made for announcements under this head, and we are always glad to receive and publish appointments. The information, to insure accuracy, should be sent from the nurses themselves, and we cannot undertake to correct official announcements which may happen to be inaccurate. It is essential that in all cases the school of training should be given.]

Borough Fever Hospital, Dover.—Miss Letitia E. Stephens has been appointed charge nurse. She was trained at Tonbridge Union Infirmary and was previously engaged in fever nursing at Mill Lane Fever Hospital, Liscard, Cheshire.

City Hospital, Coventry.—Miss Mary E. Williams has been appointed charge nurse. She was trained at the Poplar and Stepney Sick Asylum and was previously nurse at the Delaney Fever Hospital, Cheltenham, and the Hospital for Skin Diseases, Birmingham.

Gainsborough Isolation Hospital.—Miss Lavinia Scott has been appointed nurse matron and has since been nurse at the Royal Infirmary, Newcastle-on-Tyne, and matron at the Isolation Hospital at Eston.

Great Yarmouth Hospital.—Miss Janet Rice has been appointed sister. She was trained at Cumberland Infirmary, Carlisle, and has since been charge nurse at Beckett Hospital, Barnsley, and charge nurse at the Northern Infirmary, Inverness. She has also done sister's holiday duty at Stockport Infirmary.

General Hospital, Loughborough.—Miss Sarah S. Neech has been appointed sister superintendent. She was trained at Guy's Hospital, London, and the Royal Infirmary, Glasgow. She has since been matron of the Memorial Hospital, Jarrow-on-Tyne, of the Hertford British Hospital, Paris, and temporary matron of the County Hospital, Guildford.

Home and Infirmary for Sick Children, Lower Sydenham.—Miss Ray has been appointed matron. She was trained at the Great Northern Central Hospital, London, and has since been sister night superintendant at St. Mark's Hospital, City Road, London, and assistant matron at the Alexandra Hospital for Hip Disease, Queen Square, London.

Leeds Maternity Home.—Miss S. M. Edwards has been appointed matron. She was trained at Salop Infirmary, Shrewsbury, and for maternity at Clapham Maternity Hospital. She has since been charge nurse at the North Western Fever Hospital, London, sister at Chester Fever Hospital, and sister at Keighley Infirmary.

Lewisham Infirmary.—Miss Sigrid Hellesen has been appointed ward sister. She was trained at the Sussex and County Hospital, Brighton, where she has since been staff nurse and theatre sister.

Southwark Infirmary, East Dulwich.—Miss F. M. Tomkinson has been appointed night superintendent. She was trained at the North Staffordshire Infirmary, Stoke-on-Trent, where she afterwards became sister. She has also been charge nurse at Richmond Hospital and sister at Southwark Infirmary.

Vale of Clwyd Sanatorium.—Miss S. Starnell Cock has been appointed nurse matron. She was trained at Princess Alice Memorial Hospital, Eastbourne, and has since been nurse-matron.

### Presentations.

West Riding Infirmary, Middlesbrough.—Miss Clara Baldwin, sister at the West Riding Infirmary, who has just been appointed nurse matron of the Miners' Hospital at Skelton-in-Cleveland, has been presented with an ornamental brass clock from the house surgeon and nurses, and with a silver serviette ring from the maids.

### Christmas Books.

(Concluded from page 156.)

CHATTO AND WINDUS.

"Prayers," by Robert Louis Stevenson. Lovers of R. L. S. (and who can number them?) will prize the slim volume containing the prayers used by "Tusitala" at family worship at Vailima. "Maurice," by Joseph Keating (6s.), is a very powerfully-written novel, dealing with underground life in a coal-mine. There are some highly dramatic scenes in the book, and the characters are drawn with deep insight and unerring fidelity to life. Of quite another type is "The Speculations of John Steele," by Robert Barr (6s.), which plunges us into the vortex of American finance. John Steele, who rises from such small beginnings, would have made a much greater success in life had he not been so disastrously susceptible to feminine influence, and his enemies are thus enabled to accomplish his downfall. "Summer Cruising in the South Seas," by Charles Warren Stoddard (6s.). A new impression of this charming work is sure to be heartily welcomed. It has already attained to the dignity of a classic and earned the highest enconiums of many great writers. Should there remain a public to whom the book is as yet unknown we can only conjure them to lose no time in becoming acquainted with these fascinating sketches. Miss Dorothy Deakin has produced quite a novel story for us this year in "The Princess and the Kitchen-maid" (3s. 6d.). Fate has ordained that the heroine, Maud, who is only fitted to be a domestic servant, shall for the first twenty years of her life be a lady at large. Maud, however, fills this position so badly that she hails with delight a reverse of fortune, and following the instincts of her grandmother the dairymaid becomes a maid of all work in a large family, and ends by marrying a dairyman. The story is most amusing, and Maud is a model of all we could wish our servants to be.

THOMAS NELSON AND SONS.

It is very rarely that we come across so excellent a book for girls as "The Ghost of Exlea Priory," by E. L. Haverfield (5s.), good boys' books being so much more plentiful than those for their sisters. The story describes life at a girls' school, and is very interesting and realistic, the dialogue being most natural and the characters life-like and cleverly differentiated. The ghost is, however, somewhat disappointing. "The Heiress of Aylewood," by Geraldine Mockler (5s.), is an attractive book, fit to place in the hands of any young girl, and although the story is a simple one, it is by no means devoid of interest and excitement. The heroine, Ethel Dunmayne, who has just left school, is the ideal of what a girl should be; energetic, full of life and spirits, athletic to the verge of boyishness, yet reliable and womanly withal, and possessed of an irresistibly sunny nature that charms all whom she meets. So well told a tale is sure to be a favourite. "Famous Sisters of Great Men," by M. Kirlew (2s. 6d.), includes short biographies of Henriette Renan, Mary Lamb, Caroline Herschel, Dorothy Wordsworth, and Fanny Mendelssohn. The book is both interesting and instructive, and contains good illustrations. "A King's Comrade," by Charles W. Whistler (5s.), is an historical tale dealing with some of the events in the Saxon period, during the reign of Ethelbert, King of East Anglia. The story is carefully based on fact, with but little fiction interwoven, which doubles the interest for us and increases the value of the book. The illustrations are quite up to the high level of the reading matter.

T. FISHER UNWIN.

In "The Interpreters," by Margaretta Byrne (6s.), we find the theme immortalised in "Enoch Arden" reproduced. But this Enoch is quite incapable of the heroic self-sacrifice of his prototype, whence come bitter suffering for the innocent victims and—at the end—tragedy. The stage is somewhat overcrowded with players, but the plot is well worked out, and the chief characters have the power of impressing us with their reality. "The Journeys of Antonia," by Christian Dundas (6s.), is a book we can warmly recommend to all our friends. We soon fall in love with Antonia Bernard, the heroine, who, by a series of mishaps, finds herself the travelling companion of George Morrier, a well-known financier. An accident, in which Morrier temporarily loses his sight, draws them together, and the two grow perilously fond of each other; but George Morrier, alas! is married—tied to a woman who has played him false and ruined his life. Antonia forces herself to bid Morrier good-bye, and refuses to let him know her whereabouts. They meet again two or three years later, when Morrier is a widower, and we can guess the sequel. The story is a charming one; would there were more like it! "Saints in Society," by Mrs. Baillie-Saunders (6s.), is the book which won the prize in Mr. Fisher Unwin's first novel competition. It has all the freshness and originality that often graces a first book, with, at the same time, a well conceived plot and many striking characters. The central figures in the story are Mark Hading, zealot and genius, and his little Cockney wife Clo. Mark is taken in hand by a nobleman of Socialistic tendencies, and rises in the world with marvellous rapidity, dragging his wife up with him. What distinguishes this book from others of the same type is the surprising results that this change of fortune produce upon the two Hadings. The development of their characters is very subtly drawn, but the husband's unexpected degeneration strains the probabilities almost as much as his wife's wonderful transformation. It is a most engrossing book, and is certain to be widely read.

C. ARTHUR PEARSON.

"The Wallypug in the Moon," by G. E. Farrow (5s.). The Wallypug needs no introduction at this time of day, and it suffices to say that he is as amusing as ever, though we are glad to think that in this book he is a little less snubbed than of yore. His visit to the moon in company of Boy and Girlie leads to a series of the most startling experiences, which all, however, end happily. Mr. Alan Wright's illustrations are a notable feature of the book. "The Adventures of Princess Daintipet," by Mrs. George Corbett (2s. 6d.), is an enchanting fairy tale and gives us a very welcome peep into fairyland. Witch Badenough is a very real and terrible person, but it is satisfactory to think the Princess escaped her in the end. This year we have another admirable edition of "Hans Andersen's Fairy Tales" (2s.), the stories which are always new and of which our children never tire. The illustrations by A. M. Brock are quite enchanting. "John of Strathbourne," by R. D. Chetwode (2s. 6d.), is a capital tale of France in the days of Francis I.

T. C. AND E. C. JACK.

Amy Steedman has produced a beautiful book dealing with the lives of some of the Saints, carefully adapted for little children. The title is "In God's Garden" (6s.). The 16 illustrations are reproductions from Italian masterpieces, and greatly enhance the beauty of the book. The stories are admirably told, and well suited for children of all ages. There are also five dainty little volumes edited by Louie Chisholm (1s. 6d. each); they belong to the "Told to the Children" series, and the stories are culled from such sources as "The Annals of Robin Hood," "The Faerie Queene," etc. They are narrated in the simple language of those who understand the need and capacity of child-mind,

CHRISTMAS BOOKS—*continued.*

and yet frequently the author's original words are given intact, without in any way interfering with the harmony of the story. The illustrations, too, are very charming. The books are as follows: "Stories from the Life of Christ," "Stories from Shakespeare," "Stories from Robin Hood," "Stories from the Faerie Queene," and "Nursery Rhymes."

The Sunday School Union.

"Kings of the Quarter-Deck," by Arthur Temple (1s. 6d.), consists of short and interesting lives of some of our admirals. We feel sure it will commend itself to all right-minded boys, and, we hope, to girls also. All little children will be pleased with "The Child's Own Magazine" this year. It is full of amusing reading, and the illustrations are decidedly good. Nothing better has been produced this Christmas for boys than "Young England"; its richly varied contents cannot fail to suit all tastes. There are two thrilling serials, "Beyond the Frontier" and "Who Wins?" and the numerous short tales are distinctly good. The book is profusely illustrated.

Skeffington and Son.

"A Memorial of Nelson," by S. Baring Gould (2s. 6d.), is a concise and interesting narrative of the career of our great naval hero and is embellished with most excellent illustrations. "Woman Disposes," by Leoline Phillips (3s. 6d.), has very little plot and the tone is far from being a pleasant one; it is not a book for "la jeune fille."

William Heinemann.

All who have read and enjoyed "A Dog's Day" will not be disappointed in "A Gay Dog," by Cecil Alden (5s.). The pictures are irresistibly funny, and the career of the "Gay Dog" is highly amusing.

P. S. King and Son.

"Louis Wain's Annual" (1s.) is as enjoyable as ever, and in saying this we feel we have paid it the highest tribute in our power. It is a book to be strongly recommended to all cat-lovers, both old and young. The humour of Mr. Wain's mirth-provoking sketches is as fresh as ever.

David Nutt.

"Mr. Ubbledejub," by A. Thorburn (2s.) is a collection of the prettiest little fairy tales imaginable. The illustrations by M. Faraday and D. Newill are beautiful, and we envy the child who becomes the happy possessor of such a lovely gift-book.

### Christmas Novelties.

(By Our Shopping Correspondent.)

LONDON GLOVE CO.

(45 Cheapside, London, E.C.)

The choice of gifts is an especially easy one this year, the shop windows having never presented a more alluring appearance. The London Glove Co. are well to the fore in their display of pretty trifles, such as gloves, feather stoles, fans, etc. Some two-buttoned Mocha gloves in soft shades of grey, beaver, and black are very popular just now, being not too thick for smartness, and warm enough to be seasonable. Where greater warmth is appreciated, the wool-lined, fur-topped gloves in brown and black, at 2s. 10d. a pair, look deliciously cosy, as do the more ambitious grey kid gloves half-lined with squirrel and fur-topped at 4s. 11d.

GARROULD'S BAZAAR.

(E. and R. Garrould, 150-160 Edgware Road, London, W.)

Garrould's Christmas bazaar is well worth a visit this year. Every kind of toy—mechanical and otherwise—that the heart of child could desire is set out on view. A swimming frog bids fair to be first favourite, and not far behind is a little balloon worked by clockwork. For grown-ups the silver goods are specially attractive, a silver-backed hairbrush with real bristles being procurable for 5s. 11d., and beautifully arranged manicure boxes at equally low figures, to say nothing of the smaller gifts in silver offered at prices which render the hall-mark quite amazing.

CADBURY'S CHOCOLATE.

(Cadbury Bros., Ltd., Bourneville, Birmingham.)

In choosing suitable presents for one's small friends, Cadbury's chocolates are a great stand-by. This grand assortment of chocolate creams of delicate flavour are put up in pretty boxes, and though moderate in price are excellent in quality.

OTHER NOVELTIES.

The Atora Beef Suet, mentioned last week, can be obtained of all grocers and dealers, or direct from Messrs. Hugon and Co., Pendleton, Manchester.

The name of the manufacturers of 4,711 Eau de Cologne should have been given last week as Messrs. Mühlen, whose London Agent is Mr. R. J. Reuter, Well Street, Cripplegate, E.C.

### Help the Nurses to Help Themselves.

**Queen Victoria's Jubilee Institute for Nurses.**—The Institute trains nurses in district nursing, and supplies nurses to affiliated associations for the sick poor in their own homes. Applications for information should be addressed to the General Superintendent, 120 Victoria Street, S.W.

**The Junius S. Morgan Benevolent Fund.**—This is an auxiliary to the Royal National Pension Fund for Nurses. It was founded by nurses as a memorial to the late Junius S. Morgan, and raised to handsome proportions by the Morgan family and many other friends to nurses. An influential committee (many members of which are hospital matrons) supervises the investigation of claims, and devotes much care and attention to the relief of necessitous members of the Pension Fund. Secretary, Mrs. Bretland Farmer.

**The Colonial Nursing Association.**—This valuable association was founded nine years ago to supply trained nurses to the Crown Colonies and small British communities in foreign countries. Since the foundation 270 nurses and matrons have been despatched to various parts of the world, grants in aid being made where it is clearly shown to be impossible for the residents unassisted to bear entire cost of passage moneys, salaries, and maintenance. The Hon. Secretary, Miss Mowbray, Imperial Institute, S.W., will be glad to receive contributions, especially as an effort is being made just now to extend its benefits to the poorer Colonies.

**East London Nursing Society.**—The object of this society is to nurse the sick poor in East London in their own homes by means of trained resident nurses, each nurse living in the parish in which she works. In 1904 the staff of 29 nurses attended to 5,277 persons, to whom 123,218 visits were made. Annual subscriptions and donations to the general fund are asked for. Secretary, Rev. A. Atkinson, Charterhouse, London, E.C.

**"The Hospital" Convalescent Fund.**—The object of this fund is to provide rest for weary and needy workers during convalescence after illness amidst suitable surroundings, without any anxiety about ways and means. Since its establishment many tired and delicate nurses have enjoyed a much-needed change of air such as they could not possibly have secured for themselves without help. Experience has proved that it is better to let the nurses have a choice of locality rather than to send them to one settled place, and nurses are accordingly sent to all parts of the country. Contributions which would increase the field of usefulness are invited by the Hon. Secretary, care of the Editor of The Hospital.

### A Book and its Story.

IN SEARCH OF THE SIMPLE LIFE.*

In writing of the emotions working beneath the studied calm necessary to royal bearing—which in the case of "Elizabeth's" latest heroine, Princess Priscilla, have fermented from girlhood and resulted in secret rebellion against the surveillance to which she, as the daughter of a German royal house, is subjected—the author gives an amusing character sketch, presented in a manner half in jest yet deadly in earnest, which gives to all that she writes a unique charm. The heroine, Princess Priscilla, has the misfortune to be possessed of a temperament inherited from a mother, born an English Princess, which is at variance with her surroundings. She is twenty-one when the book begins, and from the age of sixteen, when she lost her mother, has lived in a state of mental unrest, not realised or understood by those around her. Her mother had suffered from an unquenchable independence of mind and a persistent intention to think which no one could stop, and, in consequence, she made her own life, and that of the members of her Court, a burden. Priscilla has learnt discretion from these characteristics of a mother untrammelled by court restrictions, and while inwardly in rebellion, bears outwardly a calm exterior, and is silent. She has two sisters far less attractive than herself. Each has married a suitable Prince, but although she has qualities which compare favourably with those of her sisters, "she had not criticised, she had not argued, she had been tractable, obedient, meek. Yet her sisters, who had often criticised and argued, and who had been rarely obedient and never meek . . ." had accepted the suitors that had been presented to them and Priscilla was single.

The reader's sympathy is asked for Princess Priscilla from the outset. "Conceive the deadly patience needed to stand passive and be talked to, amused, taken care of all day long for years. Conceive the intolerableness, if you are at all sensitive, of being watched by eyes so sharp and prying, so eager to note the least change of expression and to use the conclusions drawn for personal ends that nothing, absolutely nothing, escapes them. Priscilla's sisters took all these things as a matter of course . . . never wanted to be alone, liked being fussed over by their ladies-in-waiting. They, happy girls, had thick skins. But Priscilla was a dreamer of dreams, a poet who never wrote poems, but whose soul, though inarticulate, was none the less saturated with the desires and loves from which poems are born. She, like her sisters, had acutally known no other states; but then she dreamed of them continuously, she desired them continuously, she read of them continuously."

One person alone is aware of her secret discontent, one person who adored her with a devotion born of faithful service as the tutor of her childhood and girlhood, and still holding office as ducal librarian. The Grand Duke's idea of education for his daughters consisted in requiring that they should know a little of everything and nothing too well. At twenty-one Priscilla's education is finished, but permission is extended for some drawing lessons from the old tutor, Herr Fritzing, now promoted to the rank of Geheimarchivrath, and the two kindred spirits continue to meet. The following passage presents the contrasting points in the characters of the two men, the father and the tutor of the rebellious Princess. "The pleasantest fashion of describing the Grand Duke will be simply to say that he was in all things, both in mind and body, the exact opposite of Fritzing. Fritzing was a man who spent his time in ignoring his body and digging away at his mind. You know the bony aspect of such men. Hardly ever is there much flesh on them; and though they are often ugly enough, their spirit blazes at you out of wonderful eyes. I call him old Fritzing. To Priscilla at twenty he seemed coeval with the pyramids and kindred hoariness, while to all those persons who were sixty-one he did not seem old at all." Two things alone reconcile the soul of Herr Fritzing to the service of the Grand Duke, Priscilla and the Ducal Library. He dislikes his life in the castle at Kunitz as much as the Princess dislikes hers. To him she owes what of culture she possesses. He had opened her eyes to a horizon beyond the little world of Kunitz and its court; "with that good teacher she had wandered down the splendid walks of culture, met there the best people of all ages, communed with mighty souls, heard how they talked, saw how they lived, and none, not one talked as they lived and talked at Kunitz. . . . Imagine a girl taught to love freedom, to see the beauty of plain things, of quietness, of things appertaining to the spirit, taught to see how ignoble it is, how intensely, hopelessly vulgar it is to spend on one's bodily comforts more than is exactly necessary. . . . Imagine how such a girl, hearing these things every afternoon of her life, would be likely to regard the palace mornings and evenings, the ceremonies, the publicity, all those hours spent as though she were a celebrated picture forced everlastingly to stand in an attitude considered appropriate, and smile while she was being looked at." Priscilla's longing for freedom has been unconsciously fostered by her tutor to a greater extent than he realises. In painting the picture of delights found outside the walls of Kunitz, England was a goal to which the eyes of his pupil were directed as a country where "glories were attainable, indeed, only by the obscure such as he himself had been, and for ever impossible to those whom Fate obliges to travel in State carriages and special trains." "Now what Priscilla had seen of England had been the insides of Buckingham Palace and Windsor Castle; of all insides the most august. To and from these she had been conveyed in closed carriages and royal trains, and there was so close a family likeness between them and Kunitz that to her extreme discomfort she felt herself completely at home. Even the presence of the Countess Disthal (lady-in-waiting) had not been wanting. She, therefore, regarded this as not seeing England at all and said so. Fritzing remarked tartly that it was a way of seeing it that most people would envy her; and she was so unable to believe him that she said "Nonsense." But Priscilla's mind is made up. "One day, after many weeks of edging nearer to it, of going all round it, yet never quite touching it, she took a deep breath and told him she had determined to run away. She added an order that he was to help her. With her most grand ducal air she merely informed, ordered, and forbade. What she forbade was, of course, the betrayal of her plans. "You may choose," she said, "between the Grand Duke and myself. If you tell him I have done with you for ever." Of course he chose Priscilla. "Priscilla," we read further, "having spent her life at the top of the social ladder was now going to get down and spend the next twenty-one years at the bottom of it; the only way to do this was to run away from Kunitz and the best and nicest place to live in at the bottom would be England." Here she explained her conviction that beautiful things grow naturally round the bottom of ladders that cannot easily reach the top; flowers of love, temperance, charity, godliness, delicate things with roots that find their nourishment in common soil." In company with Herr Fritzing and her maid as escort, the princess reaches England and her adventures, disillusionments, and conquests fill the pages with witty dialogues and scenes improbable enough to be in keeping with carrying out of the royal plan for high thinking and plain living.

* "The Princess Priscilla's Fortnight." By the author of "Elizabeth and her German Garden." (Smith, Elder and Co., 6s.).

### Notes and Queries.

**REGULATIONS.**

**The Editor is always willing to answer in this column, without any fee, all reasonable questions, as soon as possible.**

**But the following rules must be carefully observed.**

1. **Every communication must be accompanied by the name and address of the writer.**
2. **The question must always bear upon nursing, directly or indirectly.**

**If an answer is required by letter a fee of half-a-crown must be enclosed with the note containing the inquiry.**

*Roman Catholic.*

(74) Can you tell me if you know of a hospital wanting probationers, and if being a Roman Catholic would make any difference?—*T. P.*

Hospitals wanting probationers generally advertise, but many of the best training schools have more applications than vacancies, so do not need to do so. A few matrons do not care for Roman Catholic probationers, but this is the exception, not the rule.

*Masseuse.*

(75) I shall be much obliged if you or any of your readers can give me any suggestions as to how I can find remunerative employment, holding a certificate for a year's training in massage and electricity. I am not averse to going abroad if the pay is likely to be better, and I speak both French and German.—*Flora.*

Advertise in The Nursing Mirror, stating exactly your qualifications. You might also write to some of the best known hydropathic establishments in England or Scotland, as they usually employ one or two masseuses.

*Phthisis.*

(76) Are there any convalescent homes (free) suitable for a young woman in quite the early stage of phthisis?—*H.*

There are very few free convalescent homes for phthisis. Write for "Medical Homes for Private Patients," published by the Scientific Press, Limited, price post free 7d., which contains a full list of secretaries; or try to gain admission for the patient into one of the hospitals for consumption.

*Surgical Work.*

(77) If "*E. P.*," whose query appeared in the issue of December 2, will send us her present address, marked "Queries," we may, through a correspondent, be able to help her to obtain the training she desires.

*Library for Medical Books.*

(78) Can you give me the address of a bookseller who will supply secondhand books for nurses, and who has a lending library?—*Nurse Smith.*

Write to Messrs. Lewis, Booksellers, Gower Street, London.

*American Nursing Paper.*

(79) Will you tell me if there is a journal in America similar to The Hospital for advertising nursing homes, etc?—*Lifu.*

The Hospital circulates in America.

"The American Journal of Nursing" or "The Trained Nurse," both published in New York, might suit. The Scientific Press, 28 & 29 Southampton Street, Strand, W.C., will supply you with copies if required.

*Maternity Nursing and Scarlet Fever.*

(80) Last July I accepted an engagement to nurse a lady in her first confinement, which was expected to take place the middle of December. A few days ago her friends sent to me saying that she had developed scarlet fever, and wished me to come and nurse her. This I refused to do, as I was engaged elsewhere almost immediately after my time was up with this case. Will you kindly tell me whether I am entitled to my fee, or, if not to the whole, whether I could claim part, and, if so, to what extent?—*L. O. S.*

We are afraid, though it bears hardly on you, that you cannot claim any part of your fee. The lady did not refuse to avail herself of your services, but, on the contrary, requested you to come and nurse her, and though, of course, you were perfectly right in refusing to attend an infectious case, there was no breach of the engagement on her part. If, however, the people are well-to-do, you might write and ask if they will not send you at least a portion of your fee. When they realise that you have not only lost the money, but your board, lodging, and washing for the month, they may decide to help you.

**Handbooks for Nurses.**

| | Post Free. |
|---|---|
| "A Handbook for Nurses." (Dr. J. K. Watson.) ... | 5s. 4d. |
| "Nurses' Pronouncing Dictionary of Medical Terms" | 2s. 0d. |
| "Art of Massage." (Creighton Hale.) ... ... ... | 6s. 0d. |
| "Surgical Bandaging and Dressings." (Johnson Smith.) ... ... ... ... ... ... ... ... ... | 2s. 0d. |
| "Hints on Tropical Fevers." (Sister Pollard.) ... | 1s. 8d. |

Of all booksellers or of The Scientific Press, Limited, 28 & 29 Southampton Street, Strand, London, W.C.

### For Reading to the Sick.

THE DIVINE COMFORTER.

Yea, the Comforter draws nigh
To the breaking, bursting heart;
For, with tender sympathy,
He has seen and felt its smart:
Through its darkest hours of ill,
He is waiting, watching still.
Dost thou ask, when comes His hour?
Then when it shall aid thee best.
Trust His faithfulness and power,
Trust in Him, and quiet rest.

Suffer on, and hope, and wait:
Jesus never comes too late.
Blessed day which hastens fast,
End of conflict and of sin!
Death itself shall die at last,
Heaven's eternal joys begin!
Then eternity shall prove
God is Light and God is Love!

*Spitta.*

If, while sickness or pain, and the nervous irritability which so often attends them, tries you, your soul cleaves steadfastly to Jesus, the Man of Sorrows, Who had "no whole part in His Body," remembering that "He Himself hath suffered that He might be able to succour us; for we have not an high priest which cannot be touched with the feeling of our infirmities," those pains will turn to rich blessings. When your heart and spirit are worn and harassed with internal struggles, which distract and hinder you, look once more to Jesus, suffering all possible mental agonies, sorrowful unto death, prostrate under His woes, bathed in bloody sweat, and ever ready to hear and share our sorrows, though we indeed "have not yet resisted unto blood, striving against sin." Such uplifting of your heart will win you strength to bear all this, and still more the crosses which seem to come straight from God's Own Hand.

Bethink you of the Mount of Olives, of Gethsemane; kneel at the foot of the Cross, and measure your spiritual desolation by that! Call to mind your dear Lord's cry of anguish, and the sorrows which beset His whole Incarnate life, and your sorrows will be more easy to bear. "He that searcheth the heart knoweth the mind of the spirit." Who knows our needs as Jesus does? Who else can fathom every depth of our heart, and see every hidden spring of thought and feeling? Will He not guide us to ask that which we really need, instead of wandering amid a crowd of petitions. It is no longer we that ask, but God's Holy Spirit asking in us; the soul becomes an instrument of His Grace: "They shall all be taught of God." We shall be sure to ask aright, because possessing Him we possess all things, and ask what we may, we shall ever rest in that blessed consciousness, knowing that He wills us to rest trustfully in Him, to seek and possess Him peacefully. "Thou wilt keep him in perfect peace whose mind is stayed on Thee, because he trusteth in Thee." *Guillore.*

Dearly were we purchased, Lord,
When Thy Blood for us was pour'd;
Think, O Christ, we are Thine own!
Hold me, guide me, as a child,
Through the battle, through the wild,
Leave me nevermore alone.

*Tersteegen.*

